# Pedestrian Trajectory Prediction in Extremely Crowded Scenarios

**DOI:** 10.3390/s19051223

**Published:** 2019-03-11

**Authors:** Xiaodan Shi, Xiaowei Shao, Zhiling Guo, Guangming Wu, Haoran Zhang, Ryosuke Shibasaki

**Affiliations:** 1 Center for Spatial Information Science, the University of Tokyo, Kashiwa 277-8568, Japan; shixiaodan@csis.u-tokyo.ac.jp (X.S.); guozhilingcc@csis.u-tokyo.ac.jp (Z.G.); huster-wgm@csis.u-tokyo.ac.jp (G.W.); zhang_ronan@csis.u-tokyo.ac.jp (H.Z.); shiba@csis.u-tokyo.ac.jp (R.S.); 2 Earth Observation Data Integration and Fusion Research Initiative, the University of Tokyo, Tokyo 153-8505, Japan

**Keywords:** trajectory prediction, human interaction, LSTM, crowded scenarios, encoder–decoder model, deep learning

## Abstract

Pedestrian trajectory prediction under crowded circumstances is a challenging problem owing to human interaction and the complexity of the trajectory pattern. Various methods have been proposed for solving this problem, ranging from traditional Bayesian analysis to Social Force model and deep learning methods. However, most existing models heavily depend on specific scenarios because the trajectory model is constructed in absolute coordinates even though the motion trajectory as well as human interaction are in relative motion. In this study, a novel trajectory prediction model is proposed to capture the relative motion of pedestrians in extremely crowded scenarios. Trajectory sequences and human interaction are first represented with relative motion and then integrated to our model to predict pedestrians’ trajectories. The proposed model is based on Long Short Term Memory (LSTM) structure and consists of an encoder and a decoder which are trained by truncated back propagation. In addition, an anisotropic neighborhood setting is proposed instead of traditional neighborhood analysis. The proposed approach is validated using trajectory data acquired at an extremely crowded train station in Tokyo, Japan. The trajectory prediction experiments demonstrated that the proposed method outperforms existing methods and is stable for predictions of varying length even when the model is trained with a controlled short trajectory sequence.

## 1. Introduction

Pedestrian trajectory prediction is a challenging, open task attracting increasing attentions owing to its potential applications in multi-object tracking, human surveillance, socio-robot navigation, and autonomous driving [1,2,3,4,5]. Although a number of related studies have appeared, this problem is far from being solved, particularly under crowded scenarios. In general, pedestrian trajectory prediction can be considered as a sequence generation problem based on the observation of past trajectories. Trajectory prediction under crowded scenarios is highly complex because it can be affected by various factors, such as trajectory pattern, human interaction, and obstacles. Among these, trajectory pattern and human interaction are considered the most crucial [6,7,8]. In the ideal case, the trajectory pattern can be obtained by the pedestrian’s walking destination. However, in the real world, it is not possible to know a pedestrian’s destination all the time. Thus, a more realistic method is to learn the trajectory pattern through past trajectory sequences. Human interaction follows certain common rules based on social etiquette [9]. Usually, a pedestrian will try to avoid collision, keep a comfortable distance from nearby strangers when they approach, and mimic partners when walking with friends as a group. However, under extremely crowded scenarios, multi-person interactions occur, which are dynamic and even more complicated.

The approaches addressing the trajectory prediction problem firstly are based on hand-crafted features. Those methods are designed manually with an eye for overcoming specific issues [10,11]. The features derived from these algorithms are hand-crafted features. Despite considerable success of old fashioned methods, they are limited to models of both sequence trajectory and human interaction, such as the Social Force model (SFM) [12], Particle filter [13], Kalman filter [14], and Gaussian process [15,16]. These methods predict the position at the next step based on the current state, and thus it is difficult to learn more trajectory cues because a long sequence of past trajectories cannot be encoded. In theory, sequence prediction can be performed through a loop, but the results rapidly degrade with prediction length. SFM is the most classic model for human interaction. It is based on certain definite rules and is limited to complex and dynamic real-world human interaction.

Deep learning is a subfield of machine learning which can learn different levels of abstraction by using hierarchical architectures [17,18,19]. Deep learning methods are highly successful recently, in particular, LSTM [20] on natural language process [21,22], which has also been introduced to trajectory prediction and has achieved exceptional performance [6,7,23,24]. LSTM can model long sequence data, unlike methods based on hand-crafted features, so that it can learn more trajectory cues, including the trajectory pattern, from past observation sequences. Originally, LSTM-based models were designed for uncrowded scenarios [25,26]. In fact, they consider all persons isolated and without any “communication” even under crowded scenarios where human interaction occurs frequently. Recently, Social LSTM, a prediction model proposed for crowded scenarios, has attracted attention. It models human interaction by pooling the latent states of all people inside a controlled neighborhood [24,27].

Most of the existing methods model motion trajectory and human interaction in absolute coordinates. Furthermore, they normalize the trajectory data and then feed the normalized data into the prediction network, which is also evaluated under a normalized scale [23]. Not only motion trajectory but also human interaction is modeled based on the normalized positions, which take values in the range [−1,1] or [0,1]. There are two reasons for that: (1) As many datasets as possible should be used to train the model. The datasets are acquired in various scenarios, and the coordinates are different. If data in real coordinates are fed to train the model directly, the model will obtain inaccurate results if the range of the test data is outside that of train data. (2) Normalized data not only stabilize the learning process but also improve the evaluation process.

Certain studies have demonstrated that both motion trajectory and human interaction are relative concepts rather than absolute [23,28]. Trajectory changes with time as pedestrians navigate themselves to the next position based on their current state. The walking speed of an average person is 0.8 m/s–1.5 m/s. Thus, the offset, which can describe the relative motion between the current position and the next position, has a certain range. The concept of offset is similar to that of Resnet [29], where performance is improved by adding residual connections. For moving persons, the major factors affecting human interaction are exactly certain critical low-level features, namely, relative walking direction and speed, and distance between two persons. To the authors’ knowledge, all existing LSTM-based methods model human interaction using absolute coordinates by pooling current-state features, as in the case of Social LSTM.

In this study, the focus is on the characteristic “relativity” of pedestrian motion, and an LSTM based data-driven architecture is proposed for trajectory prediction in extremely crowded scenarios. To capture the “relativity” of pedestrian motion, the relative motion of the trajectory sequence and human interaction is modeled using different strategies. Then, they are integrated into a trajectory prediction model. It has an encoder–decoder architecture, where both the encoder and the decoder are trained using truncated back propagation through time (TBPTT) [30,31]. The main contributions of this study are as follows:

- An LSTM based prediction model for extremely crowded scenarios is proposed that can model both motion trajectory and human interaction with relative motion.
- To the best of our knowledge, it is the first time to consider interaction by modeling relative motion among pedestrians trajectory for trajectory prediction.
- Attention-weighted pooling is used to model human interaction dynamically.

The remainder of this paper is organized as follows: in Section 2, an overview of related works is presented. In Section 3, the data source is introduced. In Section 4, the approach based on the LSTM model is described. In Section 5, the experimental results are presented. Then, the paper is concluded with contributions and suggestions for future research.

## 2. Related Works

Our main task of interest is in pedestrian trajectory prediction using recurrent neural networks (RNNs), in particular, LSTM based architectures. For long trajectory sequence prediction, RNN based methods have achieved the exceptional accuracy [6,23,24,32,33]. Thus, in this part, we will briefly review these deep learning methods.

### 2.1. Trajectory Prediction Based on RNNs

Recently, RNNs have attracted considerable attention owing to their ability to model long data sequences, and RNN based methods have achieved exceptional performance in trajectory prediction. Prior to RNNs, there were also certain methods for trajectory prediction based on hand-crafted features, such as Particle filter, Kalman filter and Gaussian process. However, it is not efficient to predict long trajectory sequences using traditional methods only. They cannot model the past sequence either. They usually calculate a transition matrix based on the current position and predict the next position. In theory, they can predict a long sequence by a loop, which implies that the prediction result is viewed as true and is then used for the next time step. However, the prediction results of those methods usually become irregular when the length of predictions becomes longer.

LSTM is the main part of RNNs. LSTM based encoder and decoder models have been used to process natural language (e.g., translation) and have been successful in modeling latent features of sequence data [22,34]. Those networks have been modified to add an attention mechanism, which is also in accordance with human cognition [21,35,36]. Humans pay different attention to different parts regardless of the activity they are involved in. The attention mechanism has improved prediction, translation, or image description models [37].

When an LSTM based encoder and decoder model is used for trajectory sequence prediction, similar with encoder and decoder Natural Language Processing models, a sequence of past trajectories is fed into the encoder and the hidden states of the last step LSTM cell in the encoder are then inputted to the decoder. The prediction result for each time instance is fed into the next LSTM cell in the decoder to realize sequence prediction. In [38], Sun et al. proposed an LSTM based encoder and decoder prediction method for laser data acquired in a care home. This method can model time-specific trajectory patterns by encoding trajectories described using absolute position and sensor rotation. In [39,40], an LSTM based trajectory predictor is developed and used for visual tracking. In these approaches, the motion model is regarded as a sequence prediction problem. The predictor provides a positive indicator for the next immediate position based on tracked sequence positions. In [41], Hug et al. evaluated the performance of an LSTM-MDL model, which contains an LSTM network and a mixture density layer (MDL), on the trajectory prediction task. Hug et al. demonstrated that the model can capture motion under complex scenarios, such as turning right or left. However, there are also some poor prediction results, particularly end-point prediction errors, which require further consideration.

### 2.2. Trajectory Prediction with Human Interaction

Trajectory prediction methods that consider human interaction will now be briefly reviewed. As mentioned earlier, human interaction is the key for prediction under crowded scenarios.

One of the earliest and most widely known methods for pedestrian trajectory prediction considering human interaction in crowded scenarios dates back to the classic SFM [12]. SFM represents a pedestrian (called agent hereafter) as a particle reacting to the energy described by the interactions with other dynamic targets and static objects such as obstacles. Based on the basic SFM, Yamaguchi et al. [42] improved the model by adding more crucial motion features, such as damping and interaction. Another widely used model is the Interacting Gaussian Process (IGP) [16] model, which represents the trajectory of an agent as a Gaussian Process. Each step of the agent is a set of Gaussian variables. IGP can represent multi-modal distributions and has relatively few parameters. The major drawback of those methods is their limited capability to model complex dynamic interaction under crowded scenarios because their performance largely depends on a set of predefined parameters, such as preferred walking speed and destinations.

The recent success of LSTM networks, which can learn long sequence cues, has led to a human trajectory prediction model called Social LSTM, which was proposed by Alahi et al. in 2016 [24]. It is the first major application of neural networks to model human interaction for pedestrian trajectory prediction under crowded scenarios, and has been the baseline for trajectory prediction problems. The main contribution of Social LSTM is that it introduces a novel pooling layer that is called “social pooling” and allows the LSTM of spatially proximal sequences to share their hidden states so that it can automatically learn typical interactions that take place among trajectories that coincide in time. Subsequently, a large number of models that can model human interaction to predict trajectory sequences were proposed. They can be classified into two groups: the first group is context–Social LSTM, which can model both human–human interaction and human–space interaction. In [8], Xue et al. proposed a hierarchical LSTM based prediction model that is called SS-LSTM and includes three different LSTMs to model individual trajectories, human interaction, and scenario layout. The model for capturing scenario layout is a convolutional neural network (CNN) stacking with LSTM that is fed with features from the CNN. In [43], Bartoli et al. modeled both human–human interaction and human–space interaction through social pooling for trajectory prediction in a museum. The second group is attention–Social LSTM, where the social pooling layer is modified as an attention-weighted pooling layer that learns different weights of neighbors to the agent. In [6], an attention mechanism was introduced into an encoder and decoder model. Fernando et al. applied soft attention to learn different weights of past sequence positions in the next position, and hard attention to capture different effects of neighbors to the agent. In [32], Vemula et al. introduced an attention–Social LSTM for social robot navigation in crowded scenarios. All the people in the scenario at any time instance are considered for calculating the influence on an agent robot. So far, the attention mechanism used in prediction has originated from natural language processing [21]. In addition to pedestrian trajectory prediction, LSTMs are also widely used for vehicle motion prediction [44,45].

Among the existing deep learning methods, only a few have modeled “relativity” of motion trajectories [23,41,46]. They use offset not position (where will the person be next) to model trajectory and achieved exceptional performance which can also demonstrate that the process of pedestrian moving can be better described by modeling “relativity” of motion trajectory. However, none of these methods take human interaction into account since it is not easy to model interaction on the premise motion trajectory is modeled with relative motion. Inspired by these methods, in this study, the “relativity” of human interaction is modeled with motion trajectory to capture more trajectory cues, thus achieving fairly high performance in extremely crowded circumstances.

## 3. Data Source

In most existing studies, pedestrian trajectory prediction is performed using image data rather than laser data [38]. In this study, we use trajectory data collected by 2D laser sensors which measure the distance of nearby objects by performing single-row scans with given height and controlled directions. With the development of multi-object tracking and social robot navigation, 2D laser sensors attract increasing attention because they are not affected by light change and can obtain the accurate location of pedestrians with less occlusion even under crowded scenarios [47,48], where occlusion is often the most serious problem for video camera based surveillance systems.

2D laser data utilized in this study was acquired at 8:00 a.m. in the lobby (approximately 60×35 m) of Osaki station in Tokyo, Japan. Eight single-row 2D laser sensors (LMS291) with a height of 20 cm were used. The original data from the sensors were fused and tracked using Particle filter. For more details on this, the reader is referred to the publication [49]. For more details about the tracked data, the reader is referred to the website [50].

**Figure 1:**
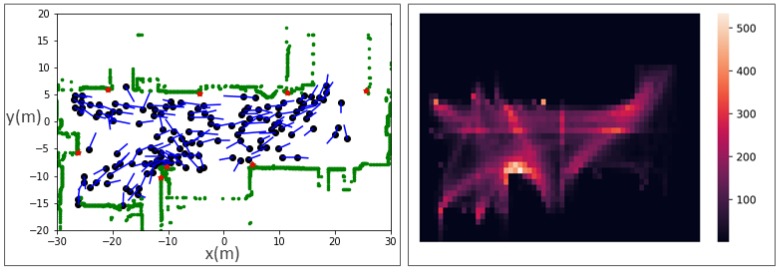
Global scenarios for the data used: (**left**) the green part is the background, each black dot is a pedestrian, the blue part is the pedestrians’ current walking phase, and red points are sensor locations; (**right**) heatmap of the scenarios. Warmer color implies higher pedestrian occupancy frequency.

The frequency of the tracked data was 20 fps. In the prediction experiment, the tracked data was sampled and the sampling rate was 2.5 fps, that is, one frame every 0.4 s. Table 1 shows the properties of data used for trajectory prediction. The train station is in the rush hour at 8:00 a.m. It is occupied with walking pedestrians. The global scenario, which is randomly selected from the data used, is shown in Figure 1 (left). In each time frame, there are on average 100 people under this scenario and 99.75% of them will stay more than 8 s. Figure 1 (right) shows the heatmap of all trajectories. Warmer color implies higher pedestrian occupancy frequency.

**Figure 2:**
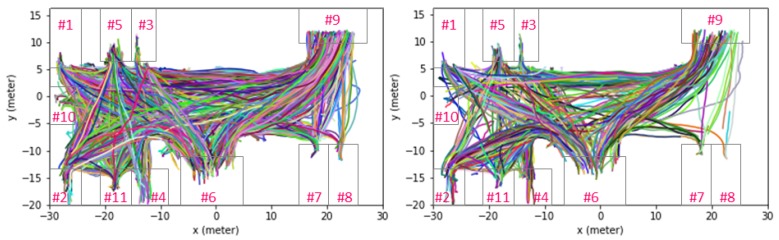
Visualization of tracklets of training and testing data: (**left**) all tracklets of training data; (**right**) all tracklets of testing data. Numbers shown in magenta are indexes of origin/destination.

There were totally 2422 trajectories under world coordinates. The entire data set was split into training data (4/5) and test data (1/5). There were 1000 frames and 1976 trajectories in the training data, and 250 frames and 446 trajectories in the testing data. The tracklets of the training and testing data are shown in Figure 2, where it can be seen that the traveling modes are complicated because there are 11 origin and exit in total at this station.

## 4. Methodology

The various motion trajectory patterns (several origins and destinations) and the dynamic human interaction are the key for a trajectory prediction model under complex circumstances. Most existing deep learning based methods heavily depend on specific scenarios because they perform trajectory prediction using absolute coordinates. In fact, the motion trajectory is relative motion coinciding with time and human interaction is relative motion among pedestrians.

This motivates the construction of a trajectory prediction model for the relative motion of both motion trajectory and human interaction. In the proposed method, the trajectory is represented with offset with respect to the last time step, and the relative motion among pedestrians is represented via a coordinate transformation. These representations are then fed into the model, which is designed with an encoder–decoder architecture. In the encoder and the decoder, a subnetwork is constructed to account for the human interaction inside an anisotropic neighborhood, and truncated back propagation through time is applied for training.

### 4.1. Brief Review on LSTM

**Figure 3:**
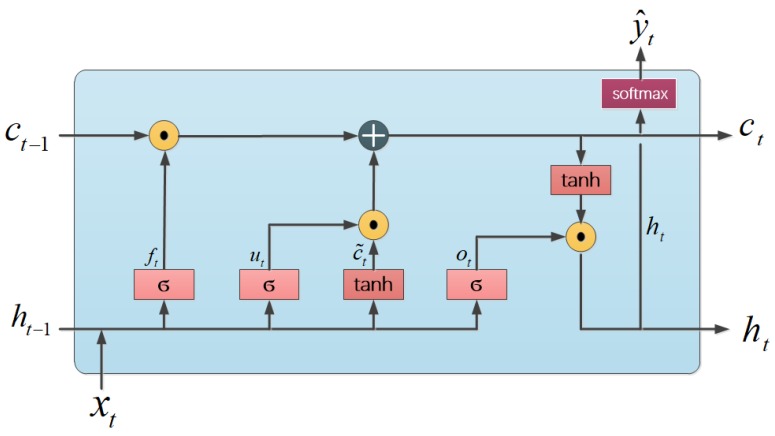
Illustration of each module of LSTM.

LSTM is introduced by Hochreiter and Schmidhuber in 1997 [20] and capable of learning long-term dependencies. LSTM contains cell states to remember the information of input sequence and gates to optionally let information through input, cell sate and output. As a special kind of RNNs, LSTM also has the form of a chain of repeating modules of neural networks. Each module of LSTM, depicted as Figure 3, works through the following equations:

(1) $$\begin{array}{cc} u_{t}\; & =\;\sigma (W_{u}\left[h_{t-1},x_{t}\right]\;+\;b_{u}),\\ f_{t}\; & =\;\sigma (W_{f}\left[h_{t-1},x_{t}\right]\;+\;b_{f}),\\ o_{t}\; & =\;\sigma (W_{o}\left[h_{t-1},x_{t}\right]\;+\;b_{o}),\\ {\tilde{c}}_{t}\; & =\;\tanh(W_{c}\left[h_{t-1},x_{t}\right]\;+\;b_{c}),\\ c_{t}\; & =\;u_{t}\;⊙\;{\tilde{c}}_{t}\;+\;f_{t}\;⊙\;c_{t-1},\\ h_{t}\; & =\;o_{t}\;⊙\;\tanh\left(c_{t}\right), \end{array}$$

where $x_{t}$ is the input vector at time instance *t*; $h_{t-1}$ and $h_{t}$ denote the hidden states at time instance t−1 and *t*; $c_{t-1}$ and $c_{t}$ are the cell states at time instance t−1 and *t*, while ${\tilde{c}}_{t}$ is a candidate cell state; ⊙ is the element-wise multiplication; $W_{u}$, $W_{f}$, $W_{o}$, $W_{c}$ are the weight matrices to calculate update gate vector $u_{t}$, forget gate vector $f_{t}$, output gate vector $o_{t}$; $b_{u}$, $b_{f}$, $b_{o}$, $b_{c}$ are the bias vectors; σ denotes a sigmoid function.

### 4.2. Problem Formulation

Trajectory prediction is viewed as a sequence generation problem, and the proposed model is based on LSTM. It is assumed that the laser data has been preprocessed, and the position and velocity of pedestrians can be obtained. At any time instance *t*, the person *i* has been described with offset ${\vec{o}}_{t}^{i}$, velocity ${\vec{v}}_{t}^{i}$, and position ${\vec{p}}_{t}^{i}$, which are expressed as follows:

(2) $$\begin{array}{cc} {\vec{p}}_{t}^{i}\; & =\;(x_{t}^{i}\;,\;y_{t}^{i}),\\ {\vec{o}}_{t}^{i}\; & =\;(ox_{t}^{i}\;,\;oy_{t}^{i}),\\ {\vec{v}}_{t}^{i}\; & =\;(vx_{t}^{i}\;,\;vy_{t}^{i}), \end{array}$$

where ${\vec{p}}_{t}^{i}$ is the position under absolute coordinate, ${\vec{o}}_{t}^{i}$ is the vector of position offset between the positions at time instances *t* and t−1, and ${\vec{v}}_{t}^{i}$ is the velocity vector.

A trajectory sequence from time $t_{0}$ to $t_{obs}$ for all persons is observed, and the future sequence from $t_{obs}$ to $t_{pred}$ is predicted. An LSTM based encoder–decoder model is constructed whose input is the sequence of observations and whose output is the sequence of predicted trajectories. Any person is treated as an agent, and all agents share the same model in this method.

### 4.3. Network Architecture

**Figure 4:**
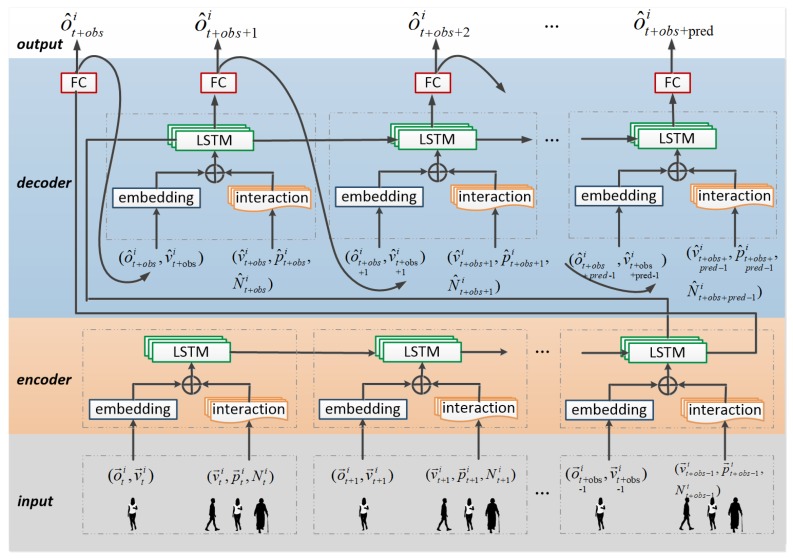
Network structure. It includes an encoder and a decoder, shown in orange and blue, respectively. The encoder encodes the observed trajectory, and the decoder performs the prediction. There is a subnetwork “interaction”, which encodes the agent–neighbor interaction.

The architecture of the proposed model is shown in Figure 4. It contains an encoder and a decoder. In the encoder, both agent’s trajectory and agent–neighbor interaction are concatenated and then fed into the LSTM. In the decoder, the output of each timestep is used to calculate the input of the next step. In this network, not only individual trajectories but also human interaction is considered. The offset ${\vec{o}}_{t}^{i}$ and the velocity ${\vec{v}}_{t}^{i}$ are used to model the agent’s past trajectory sequence, whereas the velocity ${\vec{v}}_{t}^{i}$ and the position ${\vec{p}}_{t}^{i}$ are used to model human interaction. The agent’s neighbors at time instance *t* will be represented as a set

(3) $$\begin{array}{c} N_{t}^{i}=\left\{\left({\vec{o}}_{t}^{j}\;,\;{\vec{v}}_{t}^{j}\;,\;{\vec{p}}_{t}^{j}\right)\;|\;j=\left(0,1,2,\ldots ,k-1\right)\right\}, \end{array}$$

where *j* is the index of a neighbor and *k* is the number of the neighbors of agent *i* at time instance *t*. At any time instance, every person can be treated as an agent. Each cell in the network at time instance *t* will be an LSTM cell stacking with a fully connected layer:

(4) $$\begin{array}{c} \;h\_s_{t+1}\;,\;c\_s_{t+1}\;\;=LSTM\;\left(\;\left(\;\phi_{a}\;\left(\;{\vec{o}}_{t}^{i}\;,\;{\vec{v}}_{t}^{i}\;\right)\;,\;\phi_{n}\;\left(\;{\vec{v}}_{t}^{i}\;,\;{\vec{p}}_{t}^{i}\;,\;N_{t}^{i}\;\right)\;\right),\;\;h\_s_{t}\;,\;c\_s_{t}\;\;\right), \end{array}$$

where $h\_s_{t+1}$, $c\_s_{t+1}$ and $h\_s_{t}$, $c\_s_{t}$ are the LSTM’s hidden states and cell states at time instances t+1 and *t*. $\phi_{a}$ is a layer for embedding the input of the motion trajectory of an agent, i.e., ${\vec{o}}_{t}^{i}$ and ${\vec{v}}_{t}^{i}$. $\phi_{n}$ is a subnetwork for modeling human interaction with relative motion, which will be explained in Section 4.4. These two parts are concatenated and then fed into an LSTM cell. The prediction results at time instance *t* are the sequence of *x* offsets and *y* offsets obtained by feeding $h\_s_{t+1}$ into a fully connected layer. The *x* offsets and *y* offsets are the distance along the *x* and *y* axes between the positions at time instances t+1 and *t*.

### 4.4. Human Interaction Model with Relative Motion

Pedestrians are adept at perceiving interaction from moving objects, but the underlying mechanism is still unknown. A large amount of research has been conducted on the type of visual information that drives this interaction. It has been demonstrated that the interaction between two moving persons is relative rather than absolute and relies on some critical low-level motion cues, namely, walking speed, motion direction, and distance [9,28].

**Figure 5:**
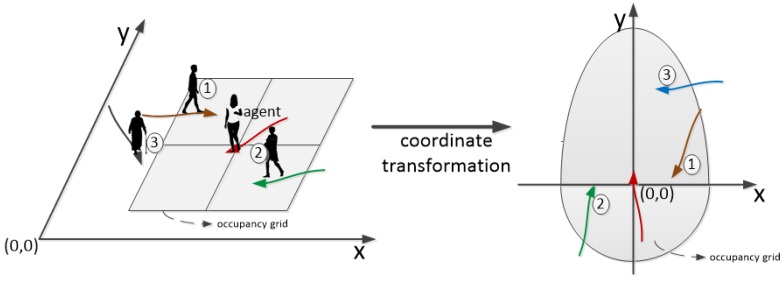
Coordinate transformation and neighborhood definition: (**left**) absolute coordinates and rectangular grid map in absolute coordinates; (**right**) relative coordinates and anisotropic neighborhood map. After a coordinate transformation, the neighbors are re-described with relative motion: relative velocity ${\tilde{v}}_{t}^{i,j}$ and relative position ${\tilde{p}}_{t}^{i,j}$.

To capture the relative motion between persons, a coordinate transformation is performed to re-describe all neighbors and re-define the neighborhood of an agent by setting the agent as the reference, as shown in Figure 5. At any time instance, the agent’s position is transformed to be located at the origin (0,0). The velocity direction of the agent always points in the vertical direction (*y*-axis). $\left({\vec{v}}_{t}^{i}\;,\;{\vec{p}}_{t}^{i}\;,\;N_{t}^{i}\right)$, the input of the subnetwork, is represented as ${\tilde{N}}_{t}^{i}\;=\;\left\{\;\left({\tilde{v}}_{t}^{i,j},\;{\tilde{p}}_{t}^{i,j}\right)\;|\;j=\left(0,1,\ldots ,k-1\right)\;\right\},$ which refers to the relative motion between agent and agent’s neighbors. ${\tilde{v}}_{t}^{i,j}$ and ${\tilde{p}}_{t}^{i,j}$ are the relative velocity and position, respectively, with respect to the agent, where ${\tilde{v}}_{t}^{i,j}\;=\;{\vec{v}}_{t}^{j}\;-\;{\vec{v}}_{t}^{i}$ and ${\tilde{p}}_{t}^{i,j}\;=\;{\vec{p}}_{t}^{j}\;-\;{\vec{p}}_{t}^{i}$.

The occupancy grid map is always used for defining the neighborhood of an agent. Rectangular grid map and circle grid map are commonly used occupancy grid maps that center at the agent’s current position [8,24,43]. Both the rectangular grid map and the circle grid map in all existing studies are designed to divide the neighborhood of an agent into grids of size m×n that are parallel to the absolute coordinate axis regardless of the agent’s walking direction. A typical rectangular grid map is shown in Figure 5 (left). Regardless of the agent’s walking direction, the grids are always parallel to the *x*- and *y*-axis. In fact, in the agent’s walking direction, the agent will look farther and pay more attention to the neighbors walking in front of him/her. Conversely, neighbors walking behind the agent will catch less attentions of the agent, and the region of interest behind the agent is smaller than that in front of him/her. To capture more neighbors who may have an influence on the agent’s trajectory in the proposed model, an anisotropic neighborhood is defined after the coordinate transformation. As shown in Figure 5 (right), the anisotropic neighborhood is the *y*-direction, is symmetrical, and is divided into two parts: the region in front of and behind the agent. The anisotropic neighborhood is defined in relative coordinates as

(5) $$R\left(x,y\right)=\left\{\begin{array}{cc} \frac{x^{2}}{a^{2}}+\frac{y^{2}}{b_{1}^{2}}=1 & \mathrm{if}\;y>=0,\\ \frac{x^{2}}{a^{2}}+\frac{y^{2}}{b_{2}^{2}}=1 & \mathrm{if}\;y<0, \end{array}\right.$$

where (x,y) is the point in relative coordinates. $a,\;b_{1},\;b_{2}$ are the parameters defining the neighborhood area. $b_{1}$ is greater than *a* and $b_{2}$ is equal to *a*, which reflects that the agent will look farther in the walking direction and pay less attention to the region behind him/her. The neighbors are grouped into two sets: in front of the agent and behind the agent. It is easy to determine which set a neighbor belongs to by calculating the angle between ${\vec{o}}_{t}^{i}$ and ${\tilde{p}}_{t}^{i,j}$.

**Figure 6:**
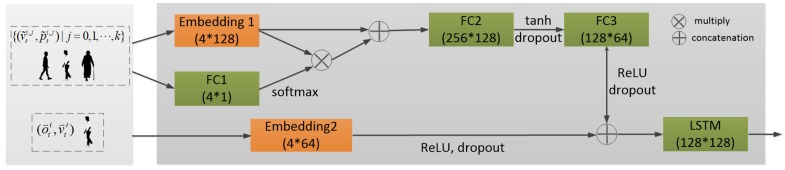
Subnetwork modeling human interaction with relative motion. FC is a fully connected layer with activation function ReLU. The first number inside the bracket is the input size of this layer and the last number is the output size. Through this subnetwork, a social tensor representing the agent–neighbor interaction can be obtained.

To model interaction with relative motion, a subnetwork is constructed as shown in Figure 6. First, an embedding layer with size 4×128 is applied to embed ${\tilde{v}}_{t}^{i,j}$ and ${\tilde{p}}_{t}^{i,j}$, and then the feature $f_{t}^{i}$ is obtained. It is worth noting that, when Social LSTM models human interaction by a social pooling layer, all neighbors are equally important to the agent, i.e., they have the same weight. However, this is not sensible, as the agent will pay different attention to different neighbors. Thus, the proposed method uses a fully connected layer to calculate the weights $w_{t}^{i}$ of the neighbors. The social tensor $s_{t}^{i}$ is computed as

(6) $$s_{t}^{i}\;=\;FC\;\left(\;FC\;\left(\;f_{t}^{i}⊗w_{t}^{i}⊕f_{t}^{i}\right)\right),$$

where ⊗ denotes tensor multiplication, and ⊕ tensor concatenation. FC denotes fully connected layers with different weights and bias, and activation function ReLU. The relative motion of the trajectory represented by offset ${\vec{o}}_{t}^{i}$ is embedded into a feature $m_{t}^{i}$ and then is fed into LSTM together with social tensor. The recurrence at time instance *t* is as follows:

(7) $$h\_s_{t+1}\;,\;c\_s_{t+1}\;=\;LSTM\left(\;\left(s_{t}^{i}⊕m_{t}^{i}\right)\;,\;h\_s_{t}\;,\;c\_s_{t}\right).$$

### 4.5. Life-Long Deployment

Given the prediction ${\hat{o}}_{t+1}^{i}$ of agent *i* at time instance *t*, the speed ${\hat{v}}_{t+1}^{i}$ and position ${\hat{p}}_{t+1}^{i}$, which are exactly the input of time instance t+1, should be estimated. Given the frequency of the sampled laser data Feq, the speed ${\hat{v}}_{t+1}^{i}$ and position ${\hat{p}}_{t+1}^{i}$ are

(8) $$\begin{array}{c} {\hat{p}}_{t+1}^{i}\;=\;{\hat{o}}_{t+1}^{i}+{\vec{p}}_{t}^{i},\\ {\hat{v}}_{t+1}^{i}\;=\;{\hat{o}}_{t+1}^{i}*Feq. \end{array}$$

The input of each time step into the decoder and the occupancy matrix are re-calculated based on the output of last time step. This process is continued until the model predicts the trajectory from $t_{obs}$ to $t_{obs+pred}$.

When the pedestrian walks in this scenario, once the observation length meets the length of the trained model, a trajectory sequence can be predicted. As trajectory length increases, new observations can be added to the model, and the eldest observation can be discarded until the pedestrian vanishes from the scenario.

### 4.6. Truncated Back Propagation through Time

The loss function used for model training is the L2 loss between prediction positions and ground truth, which is depicted as follows:

(9) $$\begin{array}{c} L_{2}\left({\hat{p}}_{t}^{i},\;{\vec{p}}_{t}^{i}\right)\;=\;\sum_{t=T1}^{T2}{∥\left({\hat{p}}_{t}^{i}-{\vec{p}}_{t}^{i}\right)∥}_{2}. \end{array}$$

**Figure 7:**
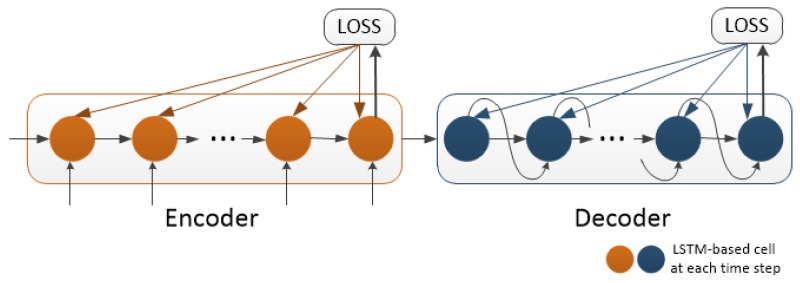
Model training using truncated back propagation through time.

Because a subnetwork is used along with the sequence prediction model, the model cannot be sufficiently trained by back propagation through time until the start of the model. The parameters of the last time step cannot be calculated correctly, which leads to zero output. Thus, the entire model is trained using truncated back propagation. As shown in Figure 7, the encoder and decoder are trained separately where T1=1, T2=obs for encoder and T1=obs+1, T2=obs+pred for decoder.

## 5. Experiments and Results

In this section, we demonstrate the effectiveness of the proposed algorithm on the extremely crowded dataset. First of all, the properties of our dataset are analyzed. Then, we report the implementation details of the experiments. Next, we compare the proposed method’s performance against other two baselines while setting various length of prediction. Two different metrics are used to measure the prediction errors. Finally, we finish the section by demonstrating some qualitative results on how the proposed approach can provide good path forecasting results.

### 5.1. Data Analysis

**Figure 8:**
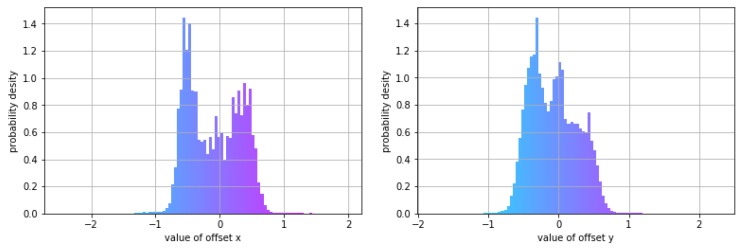
Offset histograms of data used: (**left**) *x* offset of the entire data set; (**right**) *y* offset of the entire data set.

The offset of the data, shown in Figure 8, was used to model motion trajectories. The *x* and *y* offsets of pedestrians are in the range [−1,1], which makes the model more stable.

**Figure 9:**
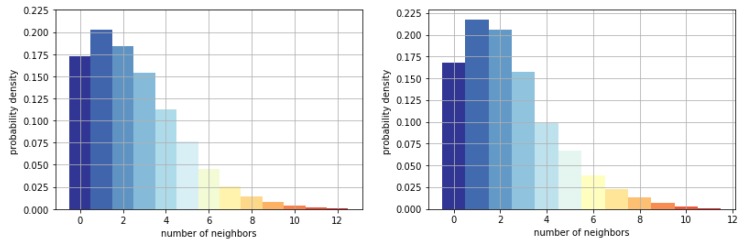
Number of neighbors in the neighborhood of agent: (**left**) person density of training data; (**right**) person density of testing data.

The parameters of the neighborhood shown in Figure 5 (right) were set as a=1, $b_{1}=2$, and $b_{2}=1$. The statistics for the number of neighbors is shown in Figure 9. In the training data, 82.75% of the pedestrians have one or more neighbors, and 44.09% have three or more. In the testing data, 83.15% of the pedestrians have one or more neighbors, and 40.81% have three or more. The data represented a highly crowded situation.

The length of data trajectories is also sufficient for the experiments. In the training data, trajectories of over 8 s are approximately 99.5% of the entire data set. In the testing data, 98.2% of the trajectories are longer than 8 s. For training, 3.2 s trajectories are observed and encoded, and then 4.8 s trajectories are predicted by the decoder. Thus, almost all the data can be used for training and testing. In reality, any trajectory length can be used for training, and trajectories with length more than the observation length can be used for testing. The model can handle the number of pedestrians dynamically both for training and testing. In training, each batch is set as a set of frames whose length is the observation length plus the prediction length, and the total number of pedestrians for this batch is obtained in advance. Thereby, there is no problem with the “birth” of a person. Furthermore, because the training loss is calculated at each time step, a person with trajectory length less than the observation and prediction length may be taken into account until the “death” of this pedestrian. In the testing phase, each person at a certain time step is set as an agent. Once the trajectory length of the agent meets the observation length, his/her next trajectory sequence is predicted. When the real observation shows that one agent disappears or appears, this person is removed or added, respectively.

### 5.2. Implementation Details

The setting of Social LSTM, Naive LSTM, and the proposed method is as follows: the code was implemented using Pytorch under Ubuntu 16.04 LTS with a GTX 1080 GPU. The truncated back propagation shown in Figure 7 was used for both the proposed method and Social LSTM.

The Social LSTM architecture that was used is the original model proposed in [24]. However, there are some differences. The input of the original Social LSTM is normalized trajectory data from images and the neighborhood size is 32 pixels. To evaluate the performance of the proposed method, Social LSTM, and Naive LSTM in real coordinates, the offset, velocity, and position of the data were input into this Social LSTM model, and the neighborhood size for the Social LSTM was set to 4 m. The parameter setting for the proposed method is a=1, $b_{1}=2$, and $b_{2}=1$.

In the implementation (including Social LSTM and Naive LSTM), only one LSTM layer was used. The parameters are shown in Table 2.

### 5.3. Evaluation Metrics

The prediction error metrics that were used are as follows:

1. Average displacement error (ADE): average L2 distance over all the prediction results and ground truth. This was introduced in [51]. ADE measures the average error of the predicted trajectory sequence.

2. Final displacement error (FDE): the distance between the prediction result and the ground truth at the final time step. FDE measures the error “destination” of the prediction. It is worth noting that both metrics were calculated in real coordinates, and thus the units of the metrics are meters.

As there are few previous studies on trajectory prediction under extremely crowded scenarios and the pedestrians’ destinations of data used are unknown, the two other prediction models that achieved state-of-the-art accuracy, namely Naive LSTM and Social LSTM, were used as the baselines for the comparison. Naive LSTM refers to the basic LSTM proposed by Hochreiter and Schmidhuber [20]. The naive LSTM treats all the trajectories to be independent from each other.

### 5.4. Experiments

We take 3.2 s trajectories as the observation (8 time steps ) and then to predict following 3.2 s (8 time steps) to 10 s (25 time steps) trajectories, respectively. The results of the proposed method and other two baselines, Naive LSTM and Social LSTM are shown in Table 3. The average computational times for training (each epoch) and testing (each trajectory) are shown in Table 4. The results from Table 3 reveal that our method can forecast path of pedestrians more accurate for any prediction length and improve the accuracy with both evaluation metrics by 21.0% than the other two baselines. However, in terms of computational cost, Naive LSTM has the best performance because it doesn’t consider the interaction among pedestrians. The proposed method has a higher computational cost than Naive LSTM and Social LSTM. It is worth noting that the proposed model is trained to predict a 4.8 s trajectory by observing a 3.2 s trajectory. However, it can also be used to predict trajectories of other lengths. From the evaluation shown in Table 3, it can be seen that Naive LSTM yields good results, and the differences among the results of the three methods are slight when the prediction length is short. As prediction length increases, the proposed method outperforms Naive LSTM and Social LSTM. Naive LSTM is not as stable as the proposed method and Social LSTM in predictions of longer length. This can be easily inferred from the final displacement error of Naive LSTM, which becomes larger as prediction length increases. The proposed method can learn more trajectory cues from short observations and can thus predict longer sequences.

### 5.5. Analysis of Trajectory Prediction Results

**Figure 10:**
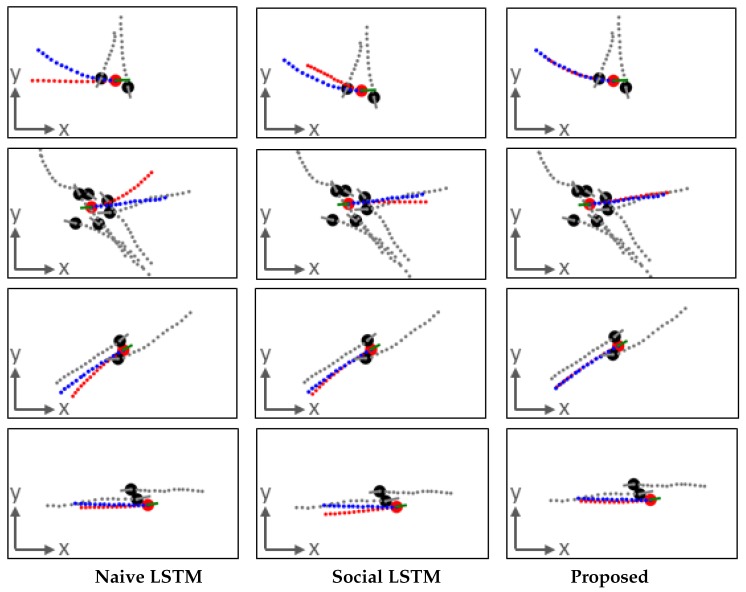
Four examples of trajectory prediction results from 3.2 s observation to 6.0 s prediction. Each row is an example. In every figure, each dot (red: agent, black: neighbors) is a pedestrian. For agent, green solid line: past trajectory, red dashed line: prediction, blue dashed line: ground truth. For neighbors, gray solid line: past trajectory, gray dashed line: following real trajectory. Results of Naive LSTM (**left**); results of Social LSTM (**middle**); results of proposed (**right**).

Examples of scenarios around some agents are now presented. The first examples in Figure 10 are from the results of a 6.0 s prediction based on a 3.2 s observation. The number of neighbors of each agent is dynamic. Each row is an example. In the first example, the agent turns slightly left to avoid the neighbor in front of him/her and then walks toward the upper-left corner. Social LSTM predicts the agent’s direction correctly but wrongly estimates the walking speed. Naive LSTM incorrectly forecasts a left walking direction. In the second example, there are several neighbors around the agent and thus various types of interaction may occur. In this case, the proposed method achieves the best performance, whereas Naive LSTM derives the wrong trajectory pattern based on the past observation. In the third example, the agent walks together with a neighbor on his/her right. Naive LSTM estimates the agent slightly off the ground truth, which is not rational because the agent adapts his/her movement to avoid collision with the neighbor on the left. In the fourth example, the proposed method and Naive LSTM yield better results than Social LSTM. This is possibly because Social LSTM considers that interaction exists between the agent and the neighbor in front of him/her. Social LSTM predicts that the agent will slightly turn left to avoid collision, but the neighbor walks faster, and thus no interaction in fact occurs in this case.

**Figure 11:**
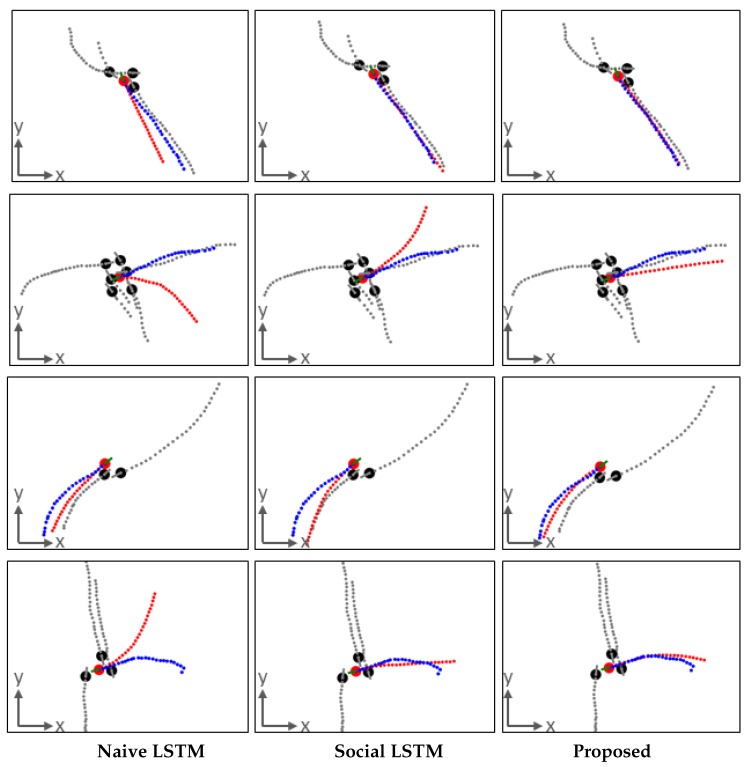
Four examples of trajectory prediction results from a 3.2 s observation to a 10.0 s prediction. Each row is an example. In every figure, each dot (red: agent, black: neighbors) is a pedestrian. For agent, green solid line: past trajectory, red dashed line: prediction, blue dashed line: ground truth. For neighbors, gray solid line: past trajectory, gray dashed line: following real trajectory. Results of Naive LSTM (**left**); results of Social LSTM (**middle**); results of proposed (**right**).

The second examples are in Figure 11. The prediction results are 10.0 s predictions from 3.2 s observations. It is interesting that the proposed method can relatively better predict the pedestrian’s trajectory for a long sequence based on a trained model on a short sequence. In the first example, both the proposed method and the Social LSTM correctly forecast the future trajectory sequence. However, the proposed method estimated the changing walking speed better than Social LSTM. In the second and fourth examples, the agent may walk in any direction. Thus, there are several possible trajectory patterns. For those cases, the proposed method can predict relatively correctly, which also demonstrates that it can learn more cues of the trajectory pattern by encoding past motion trajectory sequences and human interaction. In the third example, Social LSTM may wrongly estimate the agent’s reaction as walking over the neighbor, whereas the proposed method estimated that the agent would slightly adjust his/her movement toward the right side to avoid collision.

## 6. Conclusions

A novel trajectory prediction method under extremely crowded scenarios was proposed. The method is an encoder–decoder model based on LSTM. It can encode motion trajectory and human interaction to predict long trajectory sequences. Instead of modeling motion trajectory and human interaction in absolute coordinates, the method uses dynamic relative coordinates. Thus, more trajectory cues can be learned, and therefore the model is stable for various prediction lengths even when it is trained using short sequences. The experiments demonstrated that the proposed method outperformed the other state-of-the-art models in every single sequence and achieved an average 21.0% with both evaluation metrics, though the computational cost of our method was higher than other baselines.

Pedestrians also interact with static elements of the scenario (e.g., walls) and other moving objects (e.g., trolley cars) when walking in the crowded circumstance, which is not considered in the proposed method. In future work, we will focus on improving trajectory forecasting performance by incorporating these interactions and finding balance between prediction accuracy and computational time. We believe the consideration of “relative motion” can also help model human–space interaction and human–others interaction. In addition, we also intend to utilize more datasets (not only our 2D laser datasets but also others) to train and test our model.

## Figures and Tables

**Table 1:** Properties of data used for trajectory prediction.

Tracks	Coordinate	Avg. Path Length(s)	Framerate	Origin/Destination	Sensor	View
2422	World	19	2.5	11	2D laser	Global

**Table 2:** Implementation parameters (proposed model, Social LSTM, and Naive LSTM).

Parameters	Optimization	Learning Rate	Dropout	Gradclip	Minibatch	LSTM Size	Embedding Size
**value**	RMSprop	0.003	0.5	10	8	128	64

**Table 3:** Evaluation of prediction results.

Methods	Naive LSTM		Social LSTM		Our Method	
Measurments	ADE (m)	FDE (m)	ADE (m)	FDE (m)	ADE (m)	FDE (m)
3.2 s’ obs to 3.2 s’ pred	0.39 ± 0.0054	0.88 ± 0.0121	0.48 ± 0.0057	0.96 ± 0.0114	**0.26 ± 0.0045**	**0.76 ± 0.0098**
3.2 s’ obs to 4.0 s’ pred	0.51 ± 0.0072	1.17 ± 0.0162	0.58 ± 0.0071	1.19 ± 0.0144	**0.45 ± 0.0059**	**0.97 ± 0.0125**
3.2 s’ obs to 5.2 s’ pred	0.70 ± 0.0095	1.66 ± 0.0227	0.74 ± 0.0089	1.52 ± 0.0183	**0.59 ± 0.0077**	**1.28 ± 0.0167**
3.2 s’ obs to 6.0 s’ pred	0.83 ± 0.0115	1.98 ± 0.0276	0.83 ± 0.0102	1.72 ± 0.0206	**0.68 ± 0.0090**	**1.47 ± 0.0198**
3.2 s’ obs to 7.2 s’ pred	1.04 ± 0.0140	2.49 ± 0.0338	0.97 ± 0.0118	2.00 ± 0.0249	**0.81 ± 0.0107**	**1.78 ± 0.0243**
3.2 s’ obs to 8.0 s’ pred	1.17 ± 0.0156	2.82 ± 0.0372	1.06 ± 0.0128	2.19 ± 0.0250	**0.89 ± 0.0124**	**1.97 ± 0.0280**
3.2 s’ obs to 9.2 s’ pred	1.37 ± 0.0188	3.31 ± 0.0452	1.18 ± 0.0153	2.48 ± 0.0303	**1.01 ± 0.0152**	**2.24 ± 0.0342**
3.2 s’ obs to 10.0 s’ pred	1.50 ± 0.0213	3.63 ± 0.0507	1.27 ± 0.0169	2.69 ± 0.0343	**1.09 ± 0.0161**	**2.43 ± 0.0357**

**Table 4:** Computational cost for training(each epoch) and testing(each traj.).

Methods	Naive LSTM	Social LSTM	Our Method
Training (s)	43.18	113.75	159.81
Testing (s)	0.23	4.10	6.67
